# CCAT1 lncRNA is chromatin-retained and post-transcriptionally spliced

**DOI:** 10.1007/s00418-024-02294-w

**Published:** 2024-05-19

**Authors:** Chaya Bohrer, Eli Varon, Eldar Peretz, Gita Reinitz, Noa Kinor, David Halle, Aviram Nissan, Yaron Shav-Tal

**Affiliations:** 1 The Mina and Everard Goodman Faculty of Life Sciences and Institute of Nanotechnology, Bar-Ilan University, Ramat Gan, Israel; 2 Biochemistry Laboratory, Samson Assuta Ashdod University Hospital, Ashdod, Israel; 3 Ziv Medical Center, Safed, Israel; 4 Surgical Innovation Laboratory, The Chaim Sheba Medical Center, Tel Hashomer, Ramat Gan, Israel

**Keywords:** *MYC*, *CCAT1*, *PVT1*, Transcription site, Post-transcriptional splicing, RNA FISH

## Abstract

Super-enhancers are unique gene expression regulators widely involved in cancer development. Spread over large DNA segments, they tend to be found next to oncogenes. The super-enhancer *c-MYC* locus forms long-range chromatin looping with nearby genes, which brings the enhancer and the genes into proximity, to promote gene activation. The colon cancer-associated transcript 1 (*CCAT1*) gene, which is part of the *MYC* locus, transcribes a lncRNA that is overexpressed in colon cancer cells through activation by *MYC*. Comparing different types of cancer cell lines using RNA fluorescence in situ hybridization (RNA FISH), we detected very prominent *CCAT1* expression in HeLa cells, observed as several large *CCAT1* nuclear foci. We found that dozens of *CCAT1* transcripts accumulate on the gene locus, in addition to active transcription occurring from the gene. The accumulating transcripts are released from the chromatin during cell division. Examination of *CCAT1* lncRNA expression patterns on the single-RNA level showed that unspliced *CCAT1* transcripts are released from the gene into the nucleoplasm. Most of these unspliced transcripts were observed in proximity to the active gene but were not associated with nuclear speckles in which unspliced RNAs usually accumulate. At larger distances from the gene, the *CCAT1* transcripts appeared spliced, implying that most *CCAT1* transcripts undergo post-transcriptional splicing in the zone of the active gene. Finally, we show that unspliced *CCAT1* transcripts can be detected in the cytoplasm during splicing inhibition, which suggests that there are several *CCAT1* variants, spliced and unspliced, that the cell can recognize as suitable for export.

**Supplementary Information:**

The online version contains supplementary material available at 10.1007/s00418-024-02294-w.

## Introduction

The regulation of gene expression in eukaryotes is complex and includes different levels of regulation including epigenetic, transcriptional, translational, and post-translational control (Willemin et al. 2024; Wang et al. 2023). Enhancers are non-coding regulatory elements that enhance the transcription of associated genes when bound by specific transcription factors and are not necessarily in proximity to the gene body. Rather, they can be found upstream, downstream, or within the coding region and are brought into the vicinity of the promoter through genome folding (Deng et al. 2012). Super-enhancers (SE) are a class of regulatory sequences with enrichment for the binding of activators of transcription factors within the sequence (Pott and Lieb 2015). SEs tend to spread over large DNA regions and are found to be enriched next to genes with known oncogenic functions (Loven et al. 2013; Hnisz et al. 2013; Jia et al. 2020).

SEs can drive the expression of oncogenic long non-coding RNAs (lncRNAs) with tumor-promoting functions. For instance, the lncRNA urothelial cancer-associated 1 (*UCA1*), which is driven by a SE, is overexpressed in ovarian cancer and leads to tumorigenesis through YAP activation (Lin et al. 2019). *LINC01503* lncRNA is located at a SE, and the binding of the transcription factor TP63 to the SE locus activates the expression of *LINC01503*. This activation leads to squamous cell carcinoma development, and the lncRNA levels are correlated with the shorter survival time of patients (Xie et al. 2018). The lncRNA *LIMD1-AS1*, which is also activated by a SE, is upregulated in glioma through its activation of CDK7, which contributes to cell proliferation (Chen et al. 2023).

A well-known SE that can express various lncRNAs in different types of cancers is the SE at the *MYC* locus (Pott and Lieb 2015; Xiang et al. 2014; Iaccarino 2017). The *c-MYC* (*MYC*) oncogene is known to be upregulated in 50–60% of all tumors, while its overexpression can be achieved by a variety of mechanisms. The *MYC* gene is located at the 8q24 locus, an area containing different enhancers that are known to be involved in diverse types of cancers. The enhancers are organized in topologically associating domains (TADs), while the binding of CTCF and cohesion proteins to the TADs contributes to their stability (Pombo and Dillon 2015; Dixon et al. 2016; Dekker et al. 2023). Several studies have identified that the long-range chromatin looping at the *MYC* locus, which leads to *MYC* hyperactivation, plays a critical role in cancer progression (Huppi et al. 2012; Lancho and Herranz 2018). A major factor in *MYC* upregulation that leads to cancer development is the binding of MYC to active binding sites on nearby enhancers, which results in high transcriptional activity (Lin et al. 2012; See et al. 2022). MYC binding to enhancers activates protein-coding genes and lncRNAs, which can indirectly regulate gene expression (Wang et al. 2020b). Those ncRNAs, which are known as super-enhancer-derived ncRNAs, play a critical role in tumorigenesis, metastasis, drug resistance, and more (Peng et al. 2019; Ge et al. 2019; Lee et al. 2020).

Two of the many lncRNAs expressed from the *MYC* locus are *PVT1* and *CCAT1*. Plasmacytoma variant translocation 1 (*PVT1*) lncRNA is located on chromosome 8q24.21 and 53 kb downstream of the *MYC* locus (Parolia et al. 2018). *PVT1* is overexpressed in various types of cancer (Liu et al. 2015; Kong et al. 2015; Zhang et al. 2018; Li et al. 2024). Through its interaction with *MYC*, *PVT1* promotes progression, invasion, metastasis, and chemoradiotherapy resistance in different tumors (Shigeyasu et al. 2020; Ansari et al. 2019). Colon cancer-associated transcript 1 (*CCAT1*), also known as *CCAT1-S* or as cancer-associated region long non-coding RNA-5 (*CARLo-5*), was first discovered to be overexpressed in patients with colorectal cancer (CRC), while the highest expression was observed in the small intestine and esophagus (Nissan et al. 2012). *CCAT1* maps to chromosome 8q24.2 and contains 2795 nucleotides. The transcript has two exons and an intron that is spliced. *CCAT1* has two isoforms: *CCAT1-L* with an extended second exon, and *5L-CCAT1* with an extended 5′UTR. The dysregulation of *CCAT1* expression affects tumorigenesis and clinical manifestations such as tumor size, metastasis, invasion, and patient survival (Wang et al. 2019; Zhan and Xian 2023).

One of the reasons for *CCAT1* upregulation is its location at the SE and its interaction with *MYC*. A DNA loop between the *MYC* promoter and the enhancer locus 335 kb upstream of *MYC* (*MYC*-335) has been demonstrated (Ahmadiyeh et al. 2010). More specifically, the *CCAT1-L* gene is located 515 kb upstream of *MYC,* and *CCAT1-L* transcripts have a functional role in chromatin looping at the *MYC* locus. DNA fluorescence in situ hybridization (DNA FISH) experiments confirmed that the *MYC* gene and the *CCAT1* gene are co-localized in the tissues of CRC patients (Xiang et al. 2014). Further studies have identified high expression of *CCAT1* in breast cancer, lung cancer, osteosarcoma, and other types of cancers (Chen et al. 2016; Lai et al. 2018; Liu et al. 2019, 2023; White et al. 2014; Alaiyan et al. 2013). At present, the exact role of *CCAT1* in cancer development is unknown.

The expression of the RNAs from the *MYC* locus has not been studied on the single-molecule RNA level. We found that the *CCAT1* lncRNA is highly detectable in HeLa cells, particularly in comparison to other cell lines that are known to overexpress *CCAT1* but have less detectable transcript levels. We, therefore, examined *CCAT1* expression patterns in HeLa cells and found that the large observed foci of *CCAT1* transcription were formed due to transcript accumulation at the gene locus. In addition, we found that *CCAT1* transcripts are post-transcriptionally spliced, and this occurs shortly after their release from the gene. Interestingly, under splicing inhibition conditions, unspliced transcripts were also found in the cytoplasm, suggesting that these transcripts are a variant that the cell can recognize as suitable for export.

## Materials and methods

### Cell culture

HeLa and RKO cells were maintained in high-glucose Dulbecco’s modified Eagle’s medium (DMEM) (Biological Industries, Beit-Haemek, Israel) containing 10% fetal bovine serum (FBS; HyClone Laboratories, Logan, UT, USA), 100 IU/mL penicillin, and 100 μg/mL streptomycin (Biological Industries). Cells were grown at 37 °C and 5% CO_2_. HT-29 and HCT116 were maintained in McCoy's 5A Medium (Biological Industries) containing 10% FBS, 100 IU/mL penicillin, and 100 μg/mL streptomycin. For transcription inhibition, cells were grown on coverslips and incubated at 37 °C for 2, 3, and 4 h with either actinomycin D (ActD) (5 µg/mL, Sigma-Aldrich, Rehovot, Israel) or 5,6-dichloro-1-β-d-ribofuranosylbenzimidazole (DRB) (25 µg/mL, Sigma-Aldrich) before fixation for 20 min in 4% paraformaldehyde (PFA). For splicing inhibition, cells were grown on coverslips and incubated at 37 °C for 1 or 6 h with pladienolide B (PLB) (0.5 µM, Santa Cruz Biotechnology, Dallas, TX, USA) before fixation for 20 min in 4% PFA.

### Total RNA purification

Total RNA was produced using TRI Reagent (Sigma-Aldrich), and DNA was removed using the TURBO DNA-free kit (Invitrogen) according to the manufacturer's instructions. Synthesis of complementary DNA (cDNA) was performed using the RevertAid™ First Strand cDNA Synthesis Kit (Fermentas/Thermo Fisher Scientific), by taking 1 μg RNA for each sample. Semi-quantitative reverse transcription polymerase chain reaction (RT-PCR) was performed using an Eppendorf thermocycler amplification for 20–35 cycles (depending on the saturation level of the genes amplified) using 1 min denaturation at 94 °C, 1 min annealing at 50 °C, 1 min extension at 72 °C; and 72 °C for 10 min for final extension. The following primers were used:

CCAT1 forward: TCCATCTGGAGCATTCACTG

CCAT1 reverse: AGCCATACAGAGCCAACCTG

c-MYC forward: AATGAAAAGGCCCCCAAGGTAGTTATCC

c-MYC reverse: GTCGTTTCCGCAACAAGTCCTCTTC

PVT1 forward: GCTGTCAAAGAGGCCTGAAG

PVT1 reverse: ACATTTCCTGCTGCCGTTTT

18S forward: TGTGCCGCTAGAGGTGAAATT

18S reverse: TGGCAAATGCTTTCGCTTT

### RNA fluorescence in situ hybridization (RNA FISH)

Cells were seeded on 18 mm coverslips and fixed for 20 min in 4% PFA, then washed in 70% ethanol overnight. Coverslips were then washed twice with 10% formamide for probes purchased from Stellaris or fluorescent light-up aptamer (FLAP) probes (Tsanov et al. 2016) diluted in 4× saline-sodium citrate (SSC). For single-molecule FISH (smFISH) on endogenous transcripts, fluorescence-labeled DNA probes targeting the *c-MYC* exon sequence (570 nm, ~10 ng probe, Stellaris), *CCAT1* intron sequence (670 nm, ~10 ng probe, Stellaris), *CCAT1-L* sequence (570 nm, ~10 ng probe, FLAP), *CCAT1-5L* (570 nm, ~10 ng probe, FLAP), *PVT1* exon sequence (570 nm, ~10 ng probe, FLAP)were hybridized overnight at 37 °C in a dark chamber in 10% formamide. The next day, cells were washed twice with 10% formamide diluted in 4× SSC for 30 min at 37 °C and then washed with 1× phosphate buffered saline (PBS). To reduce photobleaching, the slides were mounted in GLOX (catalase, glucose oxidase) buffer (pH 8.1 mM, 2× SSC, 0.4% glucose) supplemented with 3.7 ng of glucose oxidase (Sigma-Aldrich G2133-10KU) and 1 μL catalase (Sigma-Aldrich 3515) prior to imaging.

### RNA FISH and immunofluorescence

For smFISH on endogenous transcripts, fluorescence-labeled DNA probes targeting the* CCAT1* exon sequence (570 nm, 10 ng, Stellaris) and the *CCAT1* intron sequence (670 nm, 10 ng, Stellaris) were hybridized overnight at 37 °C in a dark chamber in 10% formamide together with the primary antibody, anti-SRRM2 (rabbit, Abcam, ab122719). The next day, cells were washed with 10% formamide, and a secondary antibody (Alexa Fluor 488 goat anti-rabbit IgG; Abcam) was added for 30 min at 37 °C, followed by washing with 1× PBS. To reduce photobleaching, the slides were mounted in GLOX buffer.

### Quantitative RNA FISH

Following RNA FISH experiments, three-dimensional (3D) stacks of cells were acquired using a wide-field fluorescence microscope at 60× magnification. Specifically, 51 z-planes were acquired for each cell with 300 nm steps. After the acquisition, the images underwent deconvolution using Huygens software and were transferred to Imaris software (Oxford Instruments, Abingdon, UK) for image processing. In Imaris, the signal of each RNA spot was evaluated using “spot object” and the transcription site was designated using “surface object.” To calculate the number of free *CCAT1* transcripts during mitosis vs. interphase, each spot was counted, following the average calculation for each group. To measure the distances of the RNAs from the transcription site, spots in the Cy5 channel were analyzed under “shortest distance from surface.”

To calculate the number of single *CCAT1* transcripts at the transcription site, the sum of the fluorescence intensity of transcription sites was measured using the Imaris “surface tracker.” Next, the common value for the fluorescence signal of single transcripts was measured using the Imaris “spot tracker” and defined as a single RNA molecule. Then, the mean intensity of a single transcript was multiplied by the number of pixels covered by the transcription site. This value was extracted from the sum of intensity at the transcription site (TS).

$$\frac{{{\text{TS}}\;{\text{intensity}}}}{{{\text{Single}}\;{\text{transcript}}\;{\text{intensity}}}} = {\text{RNA}}\;{\text{number}}\;{\text{per}}\;{\text{TS}}$$

### Fluorescence microscopy

Wide-field fluorescence images were obtained using the cellSens system based on an Olympus IX83 fully motorized inverted microscope (60× UPlanXApo objective, 1.42 NA) fitted with a Prime BSI scientific complementary metal–oxide–semiconductor (sCMOS; Teledyne) driven by CellSens software. Images were then created using the FIJI software package.

### Statistical analysis

The experiments presented were carried out at least three times. Statistical analysis was performed using GraphPad Prism 10 software. For quantification of *CCAT1* unspliced transcripts in interphase versus mitotic cells, data were analyzed with independent-samples *t*-tests. For quantification of *CCAT1* transcripts at the transcription site, proximity to the transcription site, location of introns in the cytoplasm, percentage of transcription sites per cell, and semi-quantitative RT-PCR data were analyzed using one-way ANOVA, followed by Tukey's post hoc analysis. Treatment groups for which all values were constant (0% cells) were analyzed separately from other treatments using one-sample *t*-tests against a constant mean value of zero. Finally, a false discovery rate (FDR) correction was applied to adjust for multiple testing.

## Results

### *CCAT1* lncRNA is highly abundant in the nucleus of HeLa cells

**Fig. 1:**
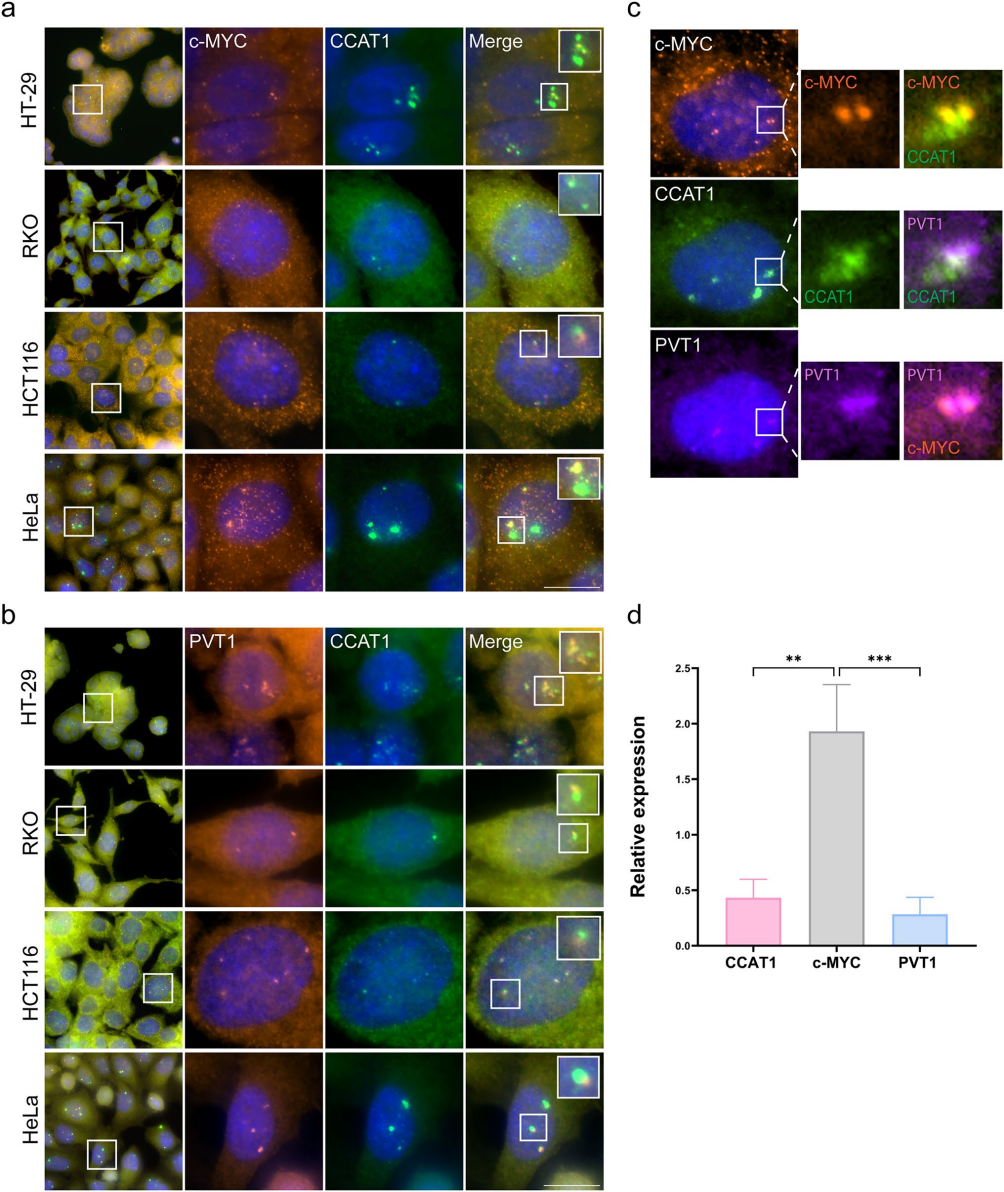
*CCAT1* lncRNA is highly abundant in nuclei of HeLa cells. Detection of **a** *CCAT1* lncRNA (exon; green) with *MYC* mRNA (orange) or with **b** *PVT1* lncRNA (orange) by RNA FISH in HT-29, RKO, HCT116, and HeLa cells. Large foci are the active genes and small dots are the single RNAs. Hoechst DNA stain is in blue. Boxed areas are enlarged. **c** RNA FISH in HeLa cells of *MYC*(orange), *CCAT1* (exon; green), and *PVT1* (purple) RNAs. Scale bars, 10 µm. **d** Expression levels of *CCAT1*,* MYC* and *PVT1* RNAs in HeLa cells measured by semi-quantitative RT-PCR. The *18S* gene was used as a housekeeping gene. Data were analyzed using one-way ANOVA, followed by Tukey's post hoc analysis. A significant difference was found in the relative expression levels between *MYC* to *CCAT1* and *PVT1*. **P < 0.01, ***P < 0.001

The *MYC* 8q24 locus has been demonstrated to express cancer-specific lncRNAs, including *CCAT1* and *PVT1* (Xiang et al. 2014; Jin et al. 2019). *CCAT1* is located 515 kb upstream of the *MYC* locus, and *PVT1* is 53 kb downstream of *MYC* (Jin et al. 2019). We examined whether the three genes could be transcriptionally active at the same time by detecting their active transcription sites using RNA FISH probes designed to hybridize with their cognate endogenous RNAs. The expression of these genes was tested in four human cancer cell lines. We tested various colorectal cancer (CRC) cell lines, as it is known that *CCAT1* is expressed in CRC: HT-29 colorectal adenocarcinoma cells, RKO colon carcinoma cells, and HCT116 colorectal carcinoma cells (McCleland et al. 2016; Yang et al. 2019; Kam et al. 2014). We also decided to include a different type of cancer cell type, HeLa cervical cancer adenocarcinoma cells (Chen et al. 2021; Li et al. 2023). As expected, the active transcription sites of *MYC* and *CCAT1* were found in all colorectal cell lines and were in close proximity (Fig. 1a). Surprisingly, HeLa cells had very large *CCAT1* transcription foci relative to the other cell lines. *MYC* mRNAs were also abundant. Testing *PVT1* lncRNA expression and *CCAT1* yielded similar results, namely that *PVT1* was expressed in all cells, and the highest signal was observed in HeLa cells (Fig. 1b). Altogether, we found that the *MYC* locus was transcriptionally active and that *CCAT1* expression seemed to be the highest of the three genes expressed from this locus. Notably for HeLa cells, the cells typically exhibited 3–4 active transcription sites expressing these genes. Indeed, chromosome 8 usually appears in three copies in HeLa cells, while chromosome translocation is also common (Landry et al. 2013). When we tested the expression of all three genes using three different probes, we found that *CCAT1*, *c-Myc*, and *PVT1* active genes were co-localized (Fig. 1c). Although *CCAT1* lncRNA showed the strongest signal at the microscopy level, we wanted to determine whether the expression levels of *CCAT1* were indeed relatively high. Semi-quantitative RT-PCR showed that *MYC* expression levels in HeLa cells were much higher than those of *CCAT1* (Fig. 1d). This finding implies that the strong *CCAT1* signals observed in the microscopy images at the gene locus do not mean that *CCAT1* is expressed at remarkably high expression levels. Rather, the lncRNA might be accumulating at the active gene locus, as it is known that many lncRNAs associate with chromatin and with their own gene loci (Calandrelli et al. 2023).

### *CCAT1* accumulates at the active gene locus

**Fig. 2:**
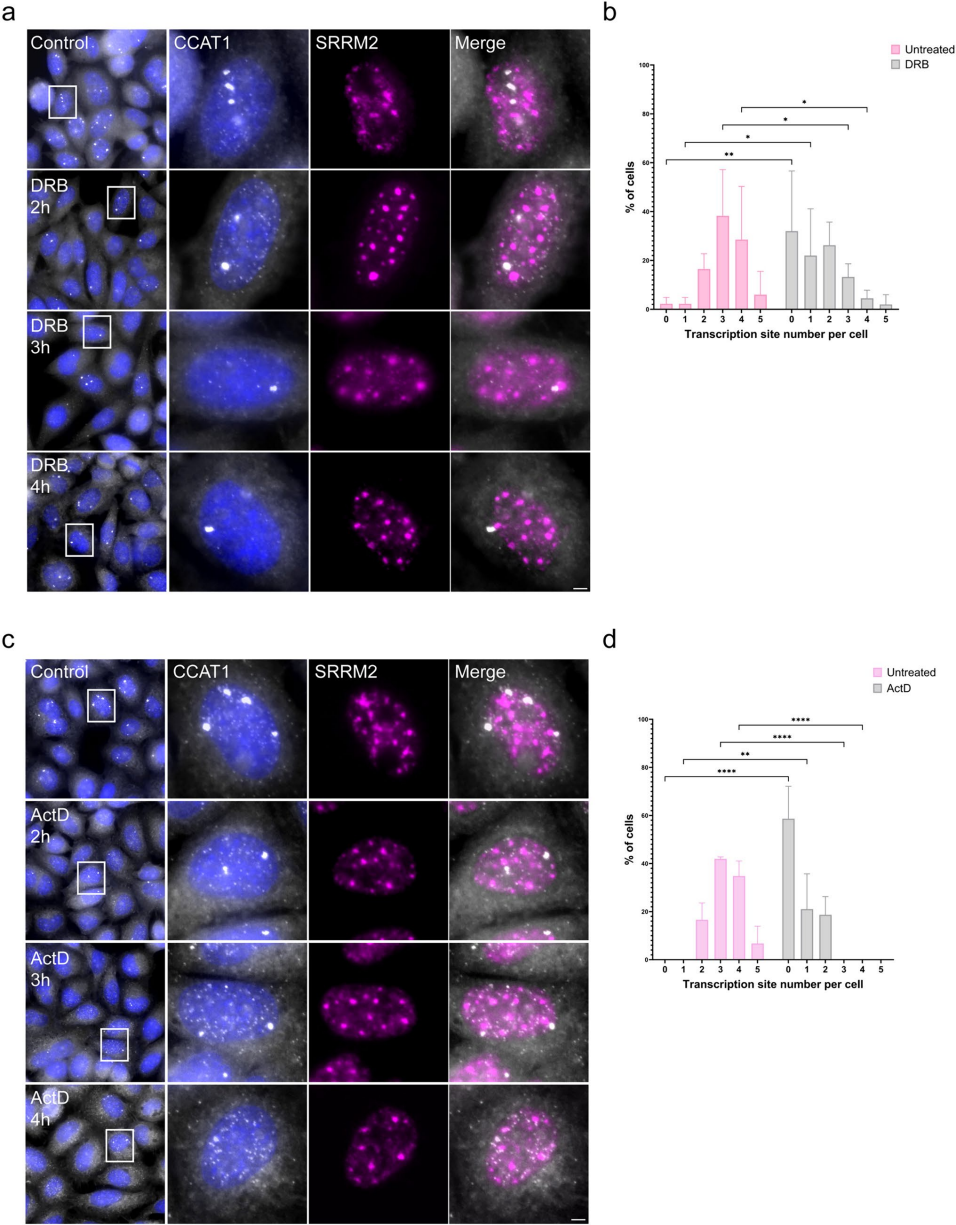
*CCAT1* lncRNAs accumulate on the gene locus. The transcription inhibitors **a** DRB or **c** ActD decreased *CCAT1* detection at the site of transcription, but small foci were still observed (4 h of treatment). *CCAT1* (exon; gray) was detected together with anti-SRRM2 (magenta) that marks nuclear speckles. Hoechst DNA stain is in blue. Boxed areas are enlarged. Scale bars, 10 µm. **b** The percentage of *CCAT1* transcription foci per cell after treatment with DRB or **d** ActD. A minimum of *n* = 50 cells were selected for each analysis. Data were analyzed using one-way ANOVA, followed by Tukey's post hoc analysis. *P < 0.05, **P < 0.01, ****P < 0.0001

To test the hypothesis that the large size of the *CCAT1* site of transcription is due to lncRNA accumulation at the locus, we examined how transcription inhibition affects the size of these transcription sites. Namely, if the enlarged size of the transcription site is due to transcript accumulation, then after treatment with a transcription inhibitor, the signal should not disappear, as would be expected from a normal active gene under transcription inhibition conditions (Brody et al. 2011; Darzacq et al. 2007). First, we used the transcription inhibitor 5,6-dichloro-1-beta-D-ribofuranosylbenzimidazole (DRB), which inhibits the CDK9 kinase (kinase subunit of positive transcription elongation factor) (Bensaude 2011) to inhibit RNA polymerase II (Pol II) transcription. Even after 4 h of treatment with DRB, *CCAT1* transcription sites were still observed (Fig. 2a). However, the percentage of visible transcription sites per cell was dramatically decreased after DRB inhibition, and ~35% of the cells did not show any visible active genes (Fig. 2b). To confirm that the DRB treatment was effective, immunofluorescence against the SRRM2 protein was performed in parallel. As expected (Rino et al. 2007; Spector and Lamond 2011), nuclear speckles that were marked by SRRM2 and that usually have an irregular shape were transformed into rounded structures due to transcription inhibition. Many genes are known to associate with nuclear speckles when transcriptionally active (Faber et al. 2022; Kim et al. 2020; Belmont 2021). However, no association between the nuclear speckles and *CCAT1* transcription sites was detected under normal or DRB-treated conditions. Similarly, when the transcription inhibitor actinomycin D (ActD) was applied, a similar but stronger effect was observed, as no visible *CCAT1* transcription sites were observed in ~60% of the cells (Fig. 2c,d). The persistence of the *CCAT1* transcription sites after transcription inhibition, rather than completely disappearing as would be expected, indicated that lncRNA accumulation was occurring at the site of transcription. Since introns are prominently detected at the site of transcription (Cote et al. 2024; Darzacq et al. 2007; Mor et al. 2010), we examined whether the intron and exon sequences of *CCAT1* overlapped at these foci. Indeed, using RNA FISH probe sets against the *CCAT1* exons or intron, we found that the intron sequences were most prominent at these foci. The latter were reduced in size when cells were treated with the transcription inhibitors (Supplementary Figure S1a, b), implying that these foci are most likely the sites at which *CCAT1* is transcribed. This means that under regular conditions, the active *CCAT1* genes produce *CCAT1* transcripts, and a subpopulation of these transcripts remains associated with the gene locus.

**Fig. 3:**
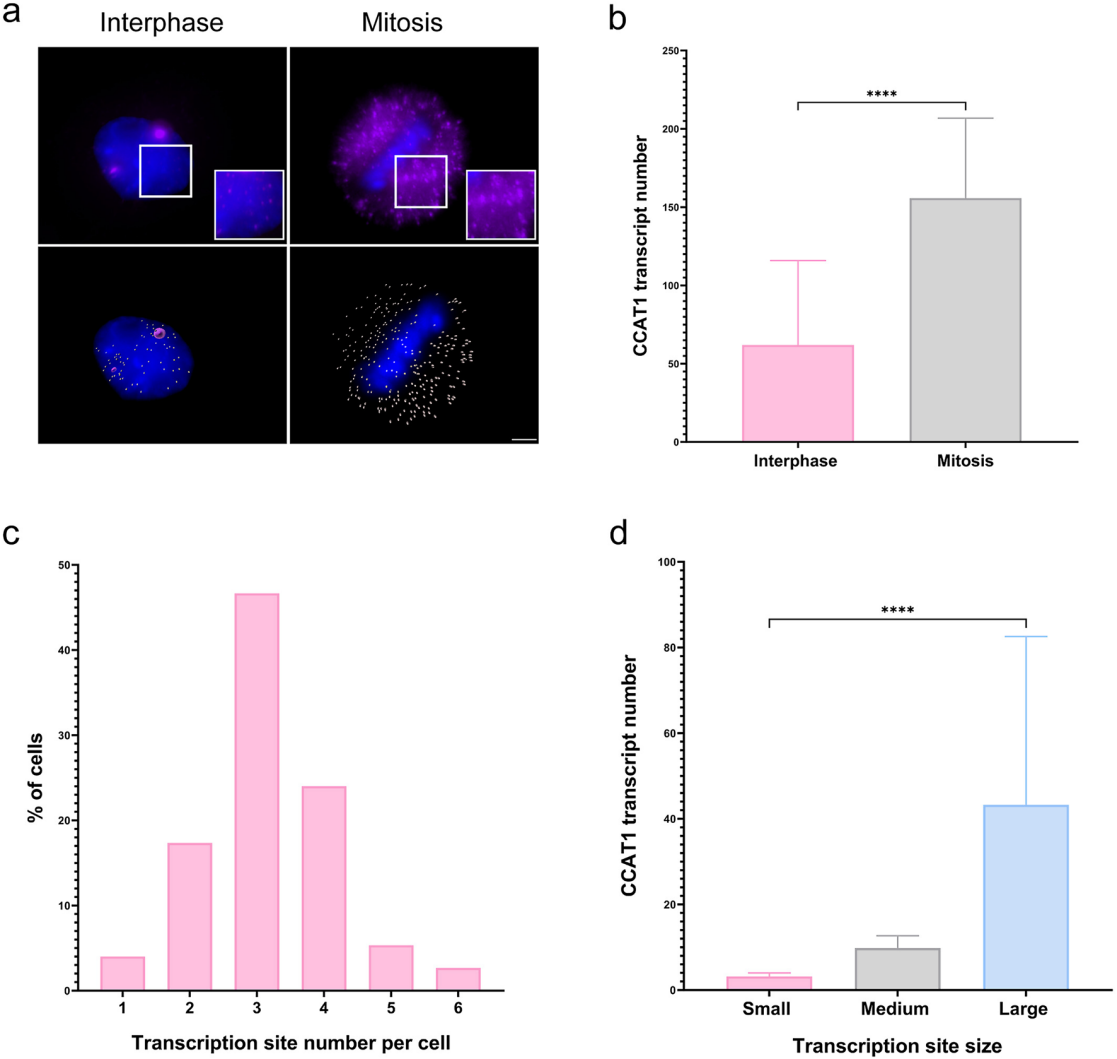
*CCAT1* transcripts are released from chromosomes during cell division. **a** Detection of *CCAT1* RNAs by RNA FISH in cells at interphase (left) and metaphase (right). The top panels show original, with zoomed images presented at high signal intensity (boxes). The bottom panel shows analyzed images (Imaris). White spots show the *CCAT1* RNA. Large pink dots are sites of transcription. Scale bar, 3 μm. **b** The total number of *CCAT1* RNAs counted in interphase versus metaphase cells. Data were analyzed with independent-samples *t*-tests. **c** The distribution of *CCAT1* transcription sites per cell under steady-state conditions.** d** Transcription foci were divided into subgroups according to their size. Each group contained different numbers of transcripts. A minimum of *n* = 50 cells were selected for each statistical analysis. ****P < 0.0001

Since we speculated that *CCAT1* transcripts were accumulating at the gene locus, we assumed that during cell division, these transcripts would dissociate from the chromosome. Also, active transcription sites were not expected to be seen since the rates of transcription decrease dramatically during cell division (Hartl et al. 1993; Palozola et al. 2017). Indeed, no *CCAT1* transcription sites were observed during cell division (Supplementary Figure S2), and no accumulation of *CCAT1* transcripts was observed on metaphase chromosomes (Fig. 3a). Rather, many *CCAT1* transcripts were now seen throughout the mitotic cell. The number of free *CCAT1* transcripts in cells during metaphase was counted and compared with the number of free transcripts in cells during interphase. A large increase in free *CCAT1* transcripts in the cell was observed (Fig. 3a, b). This indicates that during interphase, *CCAT1* transcripts accumulate at the locus, and during mitosis, they are released from the chromosomes. We could now quantify the number of *CCAT1* transcripts that were associated with the locus in cells during interphase. First, the numbers of *CCAT1* loci were counted (*n* = 300 cells), which showed that ~50% of the cells contained at least three active sites of transcription (Fig. 3c). Next, the estimated number of *CCAT1* transcripts associated with a single transcription site was calculated using the single *CCAT1* transcript average intensity (Fig. 3d). Since the *CCAT1* loci were not uniform in size, they were divided into several different subgroups. Large transcription sites contained ~40 transcripts, moderate-sized had ~10 transcripts, and the small ones contained less than 5 transcripts. Therefore, a cell that had three large loci would amount to a total of ~120 *CCAT1* transcripts associated with the gene loci. This number is within the range of the average number of *CCAT1* transcripts measured in the metaphase cells (Fig. 3b; ~150 transcripts). Altogether, these numbers correlate well, as there are also free *CCAT1* transcripts in the nucleoplasm and cytoplasm of interphase cells.

### *CCAT1* transcripts undergo post-transcriptional splicing

**Fig. 4:**
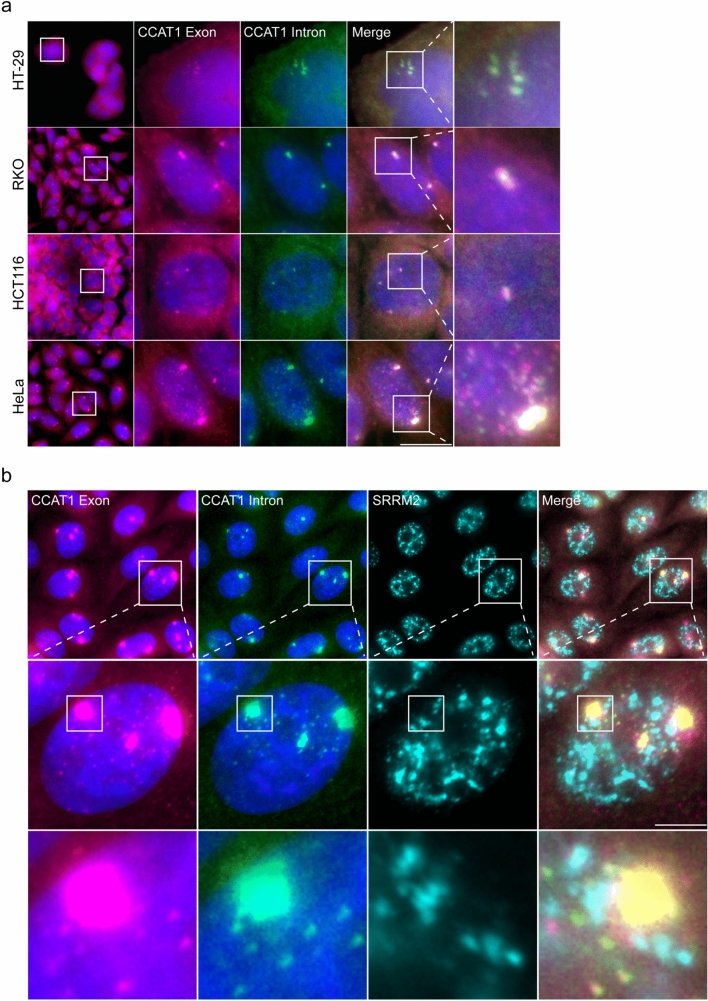
*CCAT1* unspliced transcripts are present in the nucleoplasm of HeLa cells and do not localize with nuclear speckles. **a** RNA FISH detection of *CCAT1* exon (pink) and *CCAT1* intron regions (green). HeLa cells displayed high levels of unspliced *CCAT1* transcripts at the sites of transcription and low levels of unspliced transcripts in the nucleoplasm. HT-29, HCT116, and RKO cells showed low levels of *CCAT1* introns (unspliced transcripts) at the site of transcription. **b** *CCAT1* active genes and nucleoplasmic transcripts were not associated with nuclear speckles marked by anti-SRRM2 (cyan). Hoechst DNA stain is in blue. Boxed areas are enlarged. Scale bars, 10 µm

We next wanted to examine whether the transcripts associated with the *CCAT1* gene loci were spliced transcripts in all cell lines expressing CCAT1. Using the RNA FISH probe sets against the *CCAT1* exons or intron, the intron signal overlapped with the site of transcription in HT-29, RKO, HCT116, and HeLa cells (Fig. 4a). Typically, intron signals are seen predominantly on sites of transcription, since much of the splicing occurs co-transcriptionally. Here, all transcription site signals contained intron signals, suggesting that the associated *CCAT1* lncRNAs were unspliced at this stage. However, unspliced transcripts were also detected in the nucleoplasm of the HeLa cells (Fig. 4a, Supplementary Figure S1a, b). In general, the vast majority of the *CCAT1* transcripts in the cells were spliced RNAs, and the unspliced transcripts were mostly found near the site of transcription. Unspliced transcripts were nuclear only. This was suggestive of post-transcriptional splicing occurring after release from the transcription site. Since unspliced RNAs can accumulate in nuclear speckles (Gordon et al. 2021; Mor et al. 2016), we examined whether there was an association of unspliced* CCAT1* transcripts with nuclear speckles. RNA FISH applied to *CCAT1* unspliced transcripts and staining for SRRM2, a core nuclear speckle protein, showed no association between the two (Fig. 4b).

**Fig. 5:**
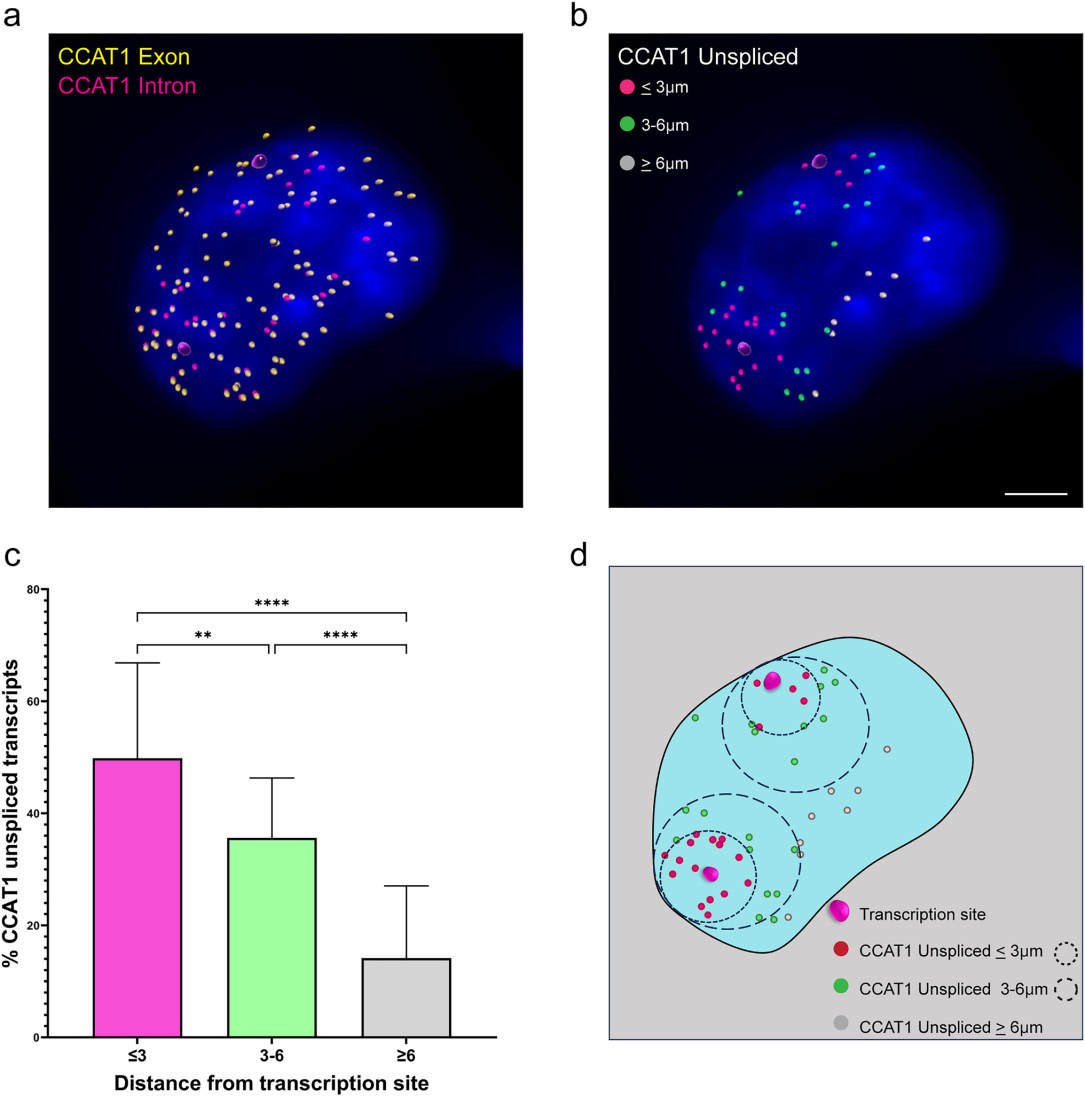
Unspliced *CCAT1* transcripts are found in close proximity to the active gene. **a** *CCAT1* spliced and unspliced RNA was detected in untreated cells and analyzed by Imaris; *CCAT1* exon spots (cyan) and *CCAT1* intron spots (red). Large pink dots are sites of transcription. **b** Distances of *CCAT1* unspliced RNAs from the active genes in untreated cells are color-coded. Pink spots show the unspliced transcripts located less than 3 μm from the active gene; green spots between 3 to 6 μm; and white spots more than 6 μm. Large pink dots are sites of transcription. **c** Measurements of the distances of *CCAT1* unspliced transcripts from the active genes. Data were analyzed with a one-way ANOVA, followed by Tukey's post hoc analysis. **P < 0.01, ****P < 0.0001. A minimum of *n* = 50 cells were selected for statistical analysis. Scale bar, 3 μm. **d** Schematic illustration of the measured distances of unspliced CCAT1 transcripts from the active genes

Next, we focused on the location of the *CCAT1* unspliced transcripts in the nucleus. Spliced *CCAT1* transcripts were dispersed over the entire nucleus, while unspliced transcripts were localized mostly next to the site of transcription (Fig. 5a). Next, we measured the distance of *CCAT1* unspliced transcripts from the gene foci (Fig. 5b). When calculating the distance of unspliced *CCAT1* transcripts from the gene locus, we found that most transcripts were located less than 3 μm from the transcription site, and less than 16% of the unspliced transcripts were located more than 6 μm from the locus (Fig. 5c). In addition, to test whether the other *CCAT1* isoforms *CCAT1-L* and *5L-CCAT1* were also post-transcriptionally spliced, RNA FISH was performed using probes that detect either *CCAT1-L* or *CCAT1-5L* only, together with probes directed to the intron. We observed that *CCAT1-L* and *CCAT-5L* expression overlapped with the intron signal in the nucleoplasm, and as observed with the *CCAT1* common isoform, the unspliced transcripts were localized close to the transcription site (Supplementary Figure S3). The presence of *CCAT1-L* and *CCAT1-5L* unspliced transcripts in proximity to the gene loci confirms that the post-transcriptional splicing is common to the three different isoforms. Taken together, these findings suggest that the splicing of *CCAT1* is post-transcriptional and occurs soon after release from the gene and before the transcripts diffuse away from the gene. This finding is consistent with the recently published evidence demonstrating that highly transcribed genes are post-transcriptionally spliced (Cote et al. 2024).

### Unspliced *CCAT1* transcripts are exported to the cytoplasm during splicing inhibition

**Fig. 6:**
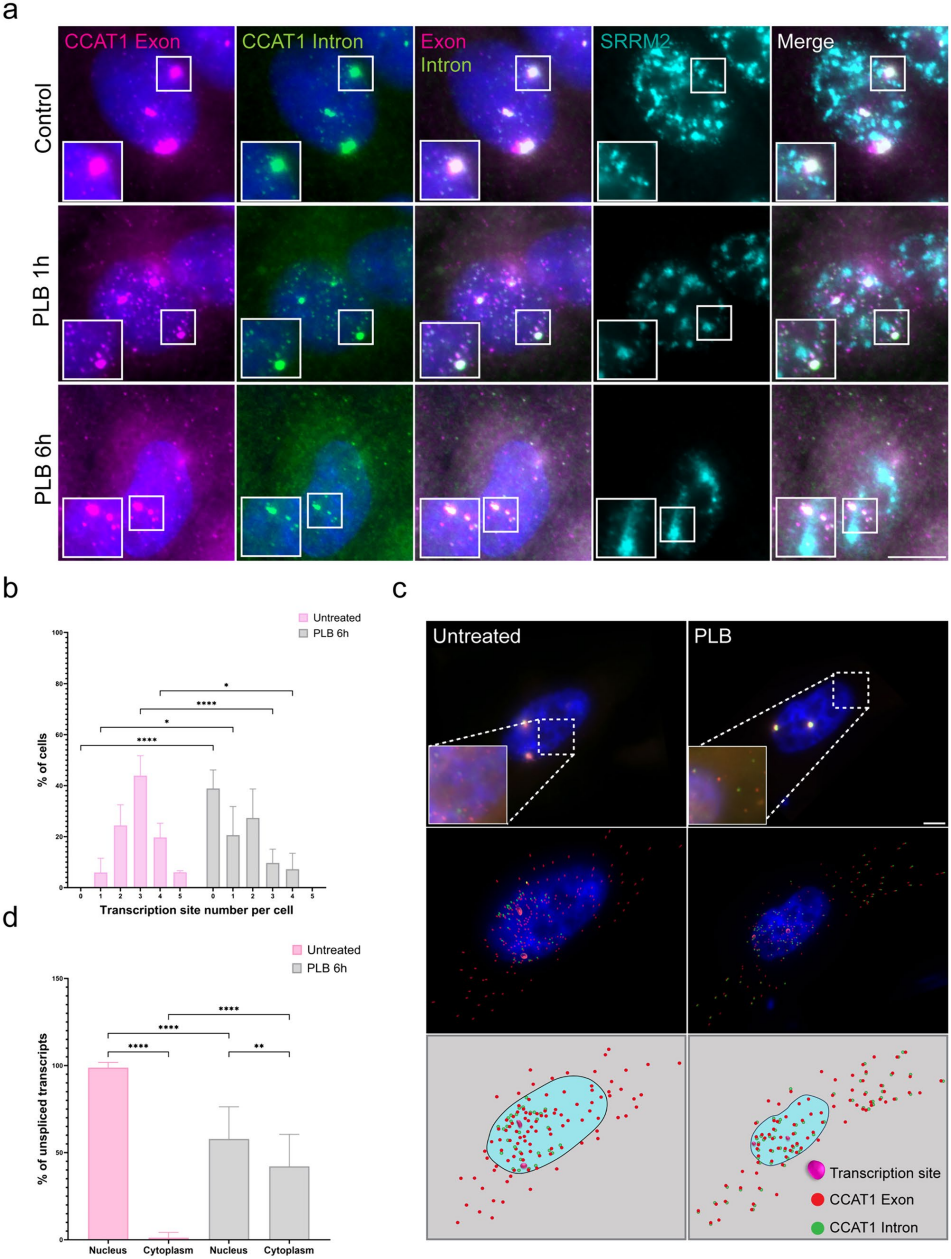
Splicing inhibition reduces *CCAT1* expression and leads to the appearance of unspliced transcripts in the cytoplasm. **a** Pladienolide B (PLB) treatment for 1 and 6 h decreased *CCAT1* detection at the active genes. Unspliced transcripts were detected at the cytoplasm after 6 h. *CCAT1* exon (pink), *CCAT1* intron (green), anti-SRRM2 for marking nuclear speckles (cyan). **b** The percentage of *CCAT1* gene foci per cell after treatment with PLB for 6 h. **c** RNA FISH of *CCAT1* exon and intron regions in untreated and splicing-inhibited conditions. *CCAT1* exon (red), *CCAT1* intron (green). The top panel represents original and zoomed images at higher intensity (boxes). The middle panel shows analyzed images (Imaris). Red spots show* CCAT1* exon and green spots show* CCAT1* intron. The bottom row presents a schematic illustration. Scale bar, 4 μm. **d** The percentage of unspliced transcripts in the cytoplasm compared with nuclei in control and under splicing inhibition conditions. Data were analyzed with **b** independent-sample *t*-tests and **d** one-way ANOVA, followed by Tukey's post hoc analysis. *P < 0.05, **P < 0.01, ****P < 0.0001. A minimum of *n* = 50 cells were selected for statistical analysis

It was previously demonstrated that splicing inhibition can lead to the accumulation of unspliced transcripts at the site of transcription and in nuclear speckles (Cote et al. 2024). These transcripts are retained in the nucleus and are not exported to the cytoplasm. We did not find any accumulation of *CCAT1* unspliced transcripts in nuclear speckles under regular conditions (Fig. 4b), and so we examined their fate under conditions of splicing inhibition. We used pladienolide B (PLB), a splicing inhibitor that binds to the SF3B1 subunit of the U2 small nuclear ribonucleoproteins (snRNP) and blocks spliceosome activity (Effenberger et al. 2017). When PLB was added to HeLa cells, the presence of *CCAT1* at the gene locus was reduced already after 1 h (Fig. 6a). After 6 h of treatment with PLB, *CCAT1* transcription sites not only did not show any visible accumulation of unspliced transcripts, but in a considerable percentage of the cells the large loci disappeared (Fig. 6b). Surprisingly, in some cells, unspliced transcripts appeared in the cytoplasm (Fig. 6a, c). As a control, we examined another transcript, the *MKI* transcript that encodes Ki-67, under splicing inhibition conditions, and unspliced *MKI* mRNAs were not detected in the cytoplasm (Supplementary Figure S4). Additionally, unspliced *CCAT1* transcripts did not appear to be associated with nuclear speckles (Fig. 6a). Quantification of the unspliced transcripts showed that ~40% of the introns were found in the cytoplasm during splicing inhibition, while the rest were in the nucleus. We postulated that following splicing inhibition, *CCAT1* transcripts, which normally are highly transcribed, undergo nuclear export despite intron retention. Taken together, these findings might suggest that *CCAT1* at steady-state conditions is post-transcriptionally spliced and that the unspliced version is not detected as an aberrant RNA with a splicing defect but might be a normal candidate for export under certain conditions.

## Discussion

Super-enhancers are large clusters of enhancers spread over large DNA regions that regulate gene expression via a variety of mechanisms. Similar to typical enhancers, SEs can be located far away from their associated gene, and in such cases, genome folding brings the enhancer into close proximity to the gene promoter, where their interactions are mediated through transcription activators and the transcriptional initiation complex, to control gene expression (Deng et al. 2012; Ye et al. 2020). SEs display enrichment of binding sites for transcription factors and activators relative to a typical enhancer (Whyte et al. 2013; Pott and Lieb 2015). SEs in cancer cells have been shown to display different expression patterns and enhancer usage relative to healthy cells (Loven et al. 2013). Therefore, SEs are considered key regulators of oncogene expression in different tumors (Tang et al. 2020).

The *MYC* locus is a tumor type-specific super-enhancer that expresses different super-enhancer-derived ncRNAs (Hnisz et al. 2013; Amjadi-Moheb et al. 2021). Within the course of our research, we focused on the expression of the *CCAT1* lncRNA at the *MYC* locus. *CCAT1* is upregulated in CRC already in the early phase of tumorigenesis as well as at late stages of the disease, and hence, *CCAT1* can be used as a potential biomarker for screening, diagnosis, and prognosis for patients with CRC (Mizrahi et al. 2015; Xiao et al. 2021). In the current study, we examined lncRNA localization in human cell lines on the single RNA level and characterized *CCAT1* expression, with an emphasis on its detection at the site of transcription in HeLa cells, which showed very prominent sites of *CCAT1* transcription. We were surprised to observe this significant upregulation of *CCAT1* in HeLa cells, since *CCAT1* is mostly correlated with colon cancer (Mizrahi et al. 2015; Shang et al. 2020). However, *CCAT1* is also expressed in ovarian cancer (Wang et al. 2020a). We found that *CCAT1* was highly detectable relative to other cell lines that express *CCAT1*; several large sites of transcription were observed and high numbers of transcripts were detected in the nucleus and cytoplasm (Fig. 1a, b). We assumed that the large foci were transcription sites since active transcription sites contain the highest number of transcripts when examined by RNA FISH, and the overlapping with the *MYC* and *PVT1* transcription sites strengthened this assumption. The relative RNA expression levels of *MYC* in HeLa cells were twofold higher than *CCAT1* levels, while the *PVT1* levels were similar to *CCAT1* (Fig. 1d).

To verify that the foci were indeed sites of transcription, we used transcription inhibitors. Following treatment with DRB or ActD, the *CCAT1* foci were still observed to a certain extent (Fig. 2a, c), although the number of cells exhibiting these foci decreased significantly. Also, the intron and exon signals predominantly co-localized at these foci, as would be expected from transcription sites that are the areas that contain most intronic sequences. The fact that the foci did not completely disappear, as would be expected of transcription sites during transcription inhibition, suggested that in addition to transcription, there is an accumulation of the lncRNAs on their gene locus. The accumulation of lncRNAs on chromatin is known (Guo et al. 2020; Schlackow et al. 2017; Calandrelli et al. 2023). The treatment with ActD had a stronger effect on transcription site disappearance (Fig. 2b, d). This effect may be due to the ActD inhibition mechanism, which functions through intercalation into GC-rich sequences and prevents RNA polymerase progression. The intercalation of ActD into the double helix may interrupt the binding of *CCAT1* to chromatin, which subsequently reduces foci size. Therefore, we speculate that the large transcription sites observed are formed due to *CCAT1* transcript accumulation on the gene locus and not due to an unusually high transcription rate modulated by RNA Pol II activity. These results correlate with previous studies showing that lncRNAs can modulate gene expression locally by accumulation at or near their site of transcription (Kopp and Mendell 2018; Gil and Ulitsky 2020). lncRNA accumulation on chromatin can be associated with its regulation or function. One of the earliest examples of RNA accumulation is the lncRNA *XIST* (Brockdorff et al. 1992; Brown et al. 1991), a key regulator of X inactivation, which mediates the silencing of the inactive X chromosome through the subsequent recruitment of epigenetic regulators (Clemson et al. 1996; Lee 2012). Another example is polyadenylated nuclear RNA (*PAN*) lncRNA, which is a viral RNA that accumulates and binds to its own promoter and robustly activates *PAN* lncRNA expression (Campbell and Izumiya 2020). Multiple studies have indicated that RNAs with processing defects, particularly in their splicing, accumulate in nuclear foci located near their site of transcription. For example, the mutant *β-globin* mRNA that has a defect in either splicing or 3′ formation has been reported to accumulate close to its site of transcription, even in the presence of transcription inhibitors, which under normal conditions cause the rapid release of wild-type human *β-globin* RNA from the vicinity of the gene (Custodio et al. 1999).

Co-transcriptional splicing of nascent RNA is a central mechanism for gene regulation in normal cells, while in some cancer cells, defective splicing machinery can shift splicing to occur post-transcriptionally (Bentley 2014). For example, a single missense mutation (Ser34Phe) in the zinc finger domain of the conserved splicing factor U2AF1 is common in different types of cancers, leading to the post-transcriptional splicing of *β*-globin and *FXR1* mRNAs (Coulon et al. 2014). A major question in the splicing field is how tightly associated are the processes of transcription and splicing. The balance between co-transcriptional and post-transcriptional splicing is regulated. Deep sequencing studies have shown that many RNAs undergo co-transcriptional splicing (Tilgner et al. 2012; Sanchez-Escabias et al. 2022). lncRNAs, however, have been reported to be less efficiently spliced than protein-coding genes, regardless of transcript activity (Mukherjee et al. 2017). One possible explanation for this phenomenon is the lack of splicing enhancer sequences at lncRNA splice sites (Krchnakova et al. 2019). Typically, splice sites are abundant along the transcribed region, while the recognition of those sites is mediated by different serine- and arginine-rich (SR) proteins and hnRNPs (De Conti et al. 2013). In HeLa cells, splicing was found to be less efficient and could occur post-transcriptionally, and in some cases the lncRNAs remain unspliced (Tilgner et al. 2012; Schlackow et al. 2017). Also, intron excision in lncRNAs is slower than protein-coding genes, and in contrast, their exon skipping is higher (Mukherjee et al. 2017). This phenomenon can be partially explained by the existence of longer lncRNA introns relative to protein-coding genes (Krchnakova et al. 2019), or also by the finding that there is less RNA Pol II pausing at transcription start sites (TES) of lncRNAs versus protein-coding genes (Schlackow et al. 2017).

A recently published study has demonstrated that highly expressed genes can undergo post-transcriptional splicing close to the transcription site zone in addition to co-transcriptional splicing which occurs during transcription (Cote et al. 2024). This study revealed that the mobility of the RNA is slower at the transcription site proximal zone than within the nucleoplasm, suggesting that the slow-moving zone is where transcripts are finally spliced. Using RNA FISH applied to the *CCAT1* exon and intron sequences revealed *CCAT1* unspliced transcripts that were located in the nucleoplasm of the cells. This was observed only in HeLa cells. The existence of unspliced transcripts was not specific to the *CCAT1* short isoform but was found for all three isoforms (Supplementary Figure S3a, b). We measured the distance that *CCAT1* unspliced transcripts traveled from the transcription site and found that most of the unspliced transcripts were localized near the site of transcription, suggesting that this transcript undergoes post-transcriptional splicing not long after release from the gene. As *CCAT1* is highly transcribed, a high number of transcripts should undergo splicing, and perhaps the delay in the splicing events led to the abundance of transcripts at the gene locus and the release of unspliced transcripts from the gene, and therefore to splicing in the nucleoplasm. This assumption is consistent with the data showing that lncRNA exhibits lower splicing rates than coding genes (Mukherjee et al. 2017). It is also possible that the accumulation of many unspliced *CCAT1* transcripts on the gene locus form a structure that sequesters the RNAs from the splicing machinery. Only after release into the nucleoplasm can the spliceosome function on these transcripts. This scenario agrees with the observation of unspliced *CCAT1* transcripts only in the region close to the gene. As mentioned, lncRNAs are less efficiently spliced since their exons contain fewer putative binding sites for SR proteins, and hence cannot generate the cooperative network of positive signals that is needed for recruiting the spliceosome to splice sites. Therefore, their splicing would be more dependent on how optimal their splice sites were (Krchnakova et al. 2019). However, the removal of introns from enhancer lncRNAs did not change their enhancing activity, meaning that introns are not essential for the activating function of lncRNAs. We find that the majority of *CCAT1* transcripts are co-transcriptionally spliced, while the minority are post-transcriptionally spliced. This can result from inefficient splicing at the site of transcription, caused by weak splicing factor interactions with the *CCAT1* transcripts.

When the cells were treated with splicing inhibitors, there was a significant decrease in *CCAT1* accumulation at the transcription site, and surprisingly, unspliced transcripts were observed in the cytoplasm of some cells. This is a rare occurrence, since introns are non-coding sequences that do not reach the cytoplasm. Moreover, when splicing is inhibited, the unspliced transcripts are usually retained at the site of transcription or accumulate in nuclear speckles (Hasenson et al. 2022; Barutcu et al. 2022; Hall et al. 2006; Johnson et al. 2000; Mor et al. 2016). These transcripts do not exit the nucleus since they are detected as aberrant transcripts. The fact that unspliced versions of *CCAT1* could be found in the nucleoplasm under normal conditions, and even in the cytoplasm under conditions of splicing inhibition, suggests that the unspliced versions are not defective but are recognized as legitimate variants and might serve roles that await discovery.

Although we do not know to what extent *CCAT1*-regulated looping engages in crosstalk with other aspects of *MYC* regulation, this study represents yet another component of the complicated *MYC* region. Our results indicate that the unusual *CCAT1* transcription site size at the gene locus is due to the accumulation of *CCAT1* transcripts, and not to exceptionally high transcription levels. This may suggest that a function of *CCAT1* transcripts on the gene locus is in the maintenance of the long-range chromatin interactions at the *MYC* locus. This shows that the accumulation of *CCAT1* at its site of transcription is poised to act as a modulator of gene expression in a locus-specific manner. Finally, as this region also expresses distinct lncRNAs in other types of human cancers, it will be of interest to learn whether other 8q24 lncRNAs behave similarly to *CCAT1*.

### Supplementary Information

Below is the link to the electronic supplementary material.

Supplementary file1 (PDF 583 KB)

## Data Availability

The datasets generated during and/or analyzed during the current study are available from the corresponding author on reasonable request.
