# Supplementary material for: CCAT1 lncRNA is chromatin-retained and post-transcriptionally spliced
Source: Histochem Cell Biol. 2024 May 19;162(1-2):91–107. doi: 10.1007/s00418-024-02294-w (PMC11227459; doi:10.1007/s00418-024-02294-w)
Supplement: Supplementary file 1 — Supplementary file1 (PDF 583 KB) [file 418_2024_2294_MOESM1_ESM.pdf]

Supplementary figures

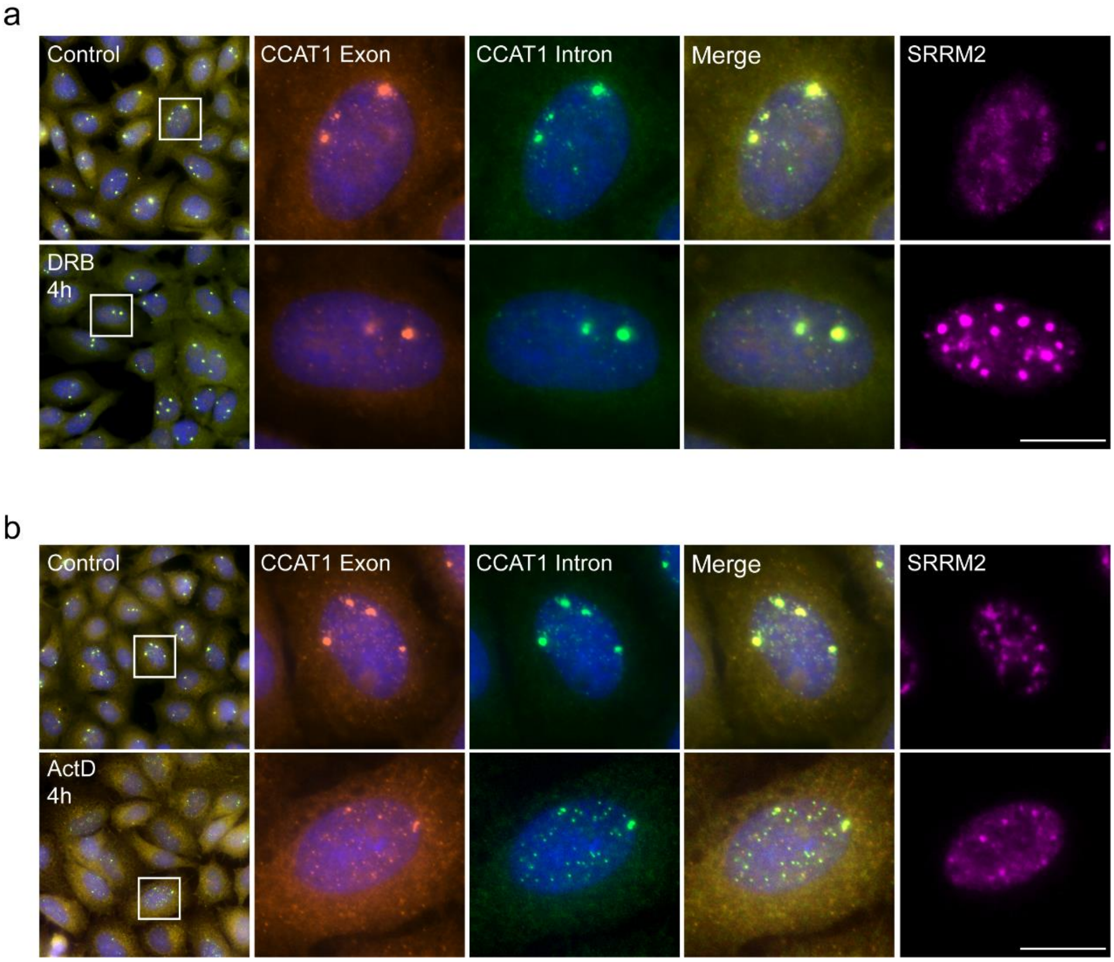

Fig. S1 *CCAT1* lncRNAs accumulate on the gene locus. RNA FISH to detect the *CCAT1* site of transcription using probes against the intron sequence. The transcription inhibitors **a** DRB or **b** ActD decreased *CCAT1* detection at the site of transcription, but small foci were still observed (4 hrs of treatment). *CCAT1* (exon; orange) and (intron; green) were detected together with anti-SRRM2 (magenta) which marks nuclear speckles. Hoechst DNA stain is in blue. Boxed areas are enlarged. Scale bars, 10 µm.

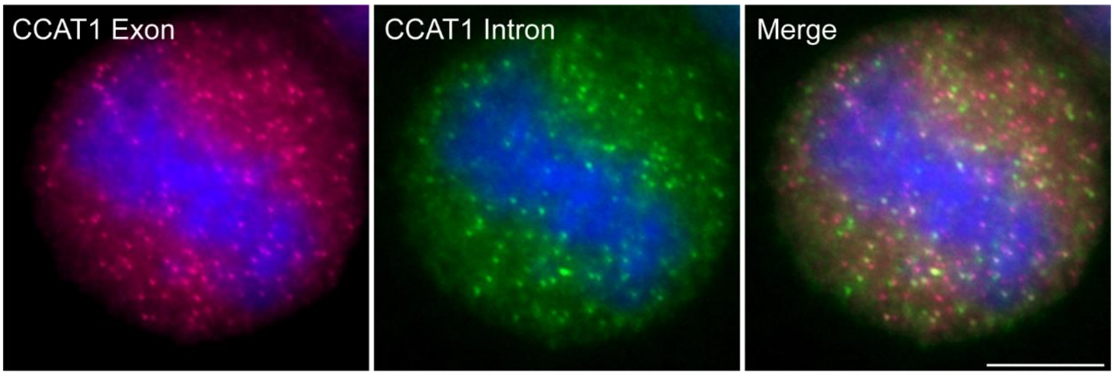

Fig. S2 *CCAT1* active genes disappear during mitosis. RNA FISH detection of *CCAT1* exon (pink), *CCAT1* intron (green). Hoechst DNA stain is in blue. Scale bar, 10 µm.

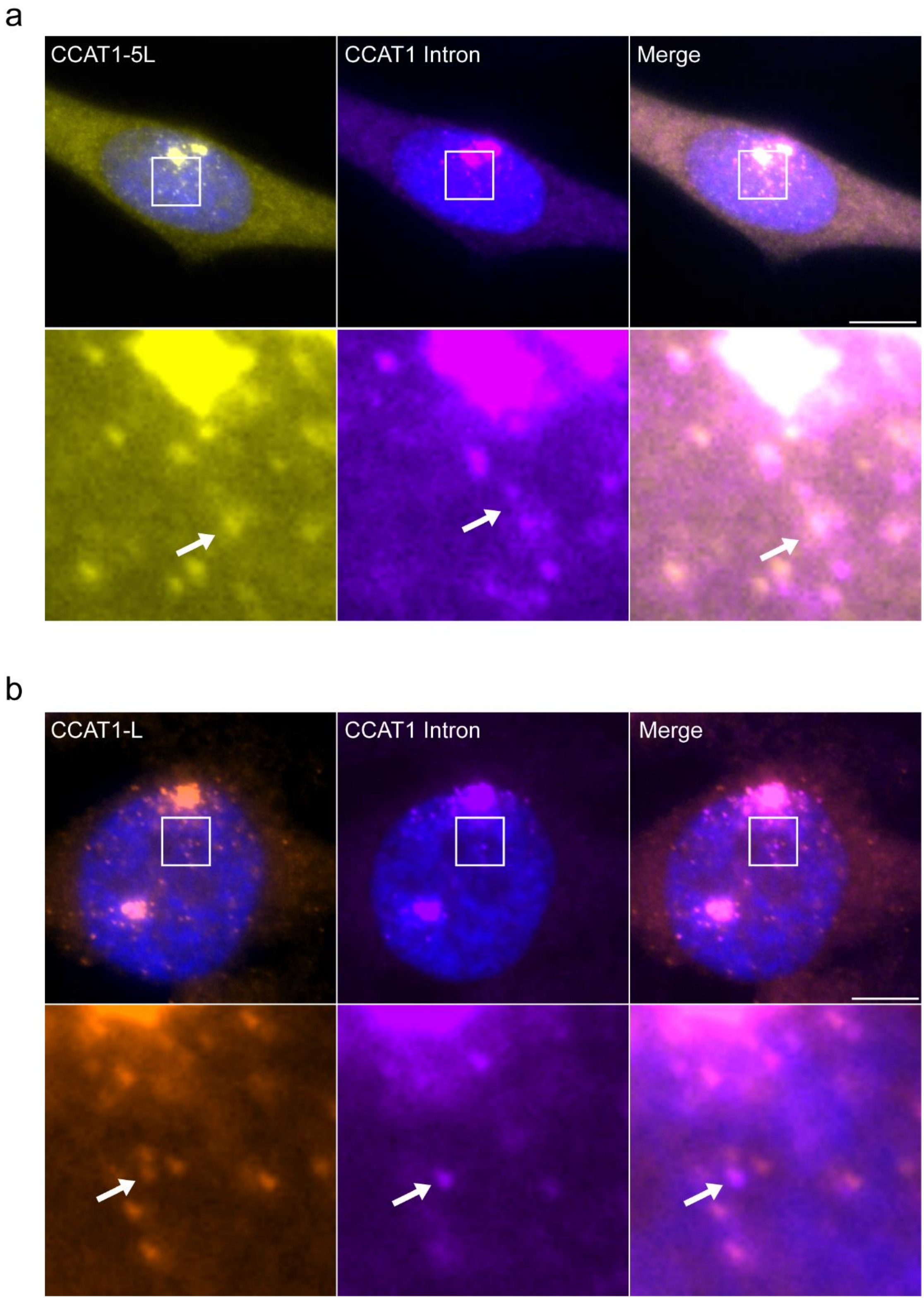

Fig. S3 All three *CCAT1* isoforms undergo post-transcriptional splicing. RNA FISH detection of **a** *CCAT1-5L* (yellow), *CCAT1* intron (purple), and **b** *CCAT1-L* (orange), shows *CCAT1-5L* and *CCAT1-L* unspliced transcripts in the nucleoplasm. Hoechst DNA stain is in blue. White arrows point to unspliced transcripts. Bar = 10 µm.

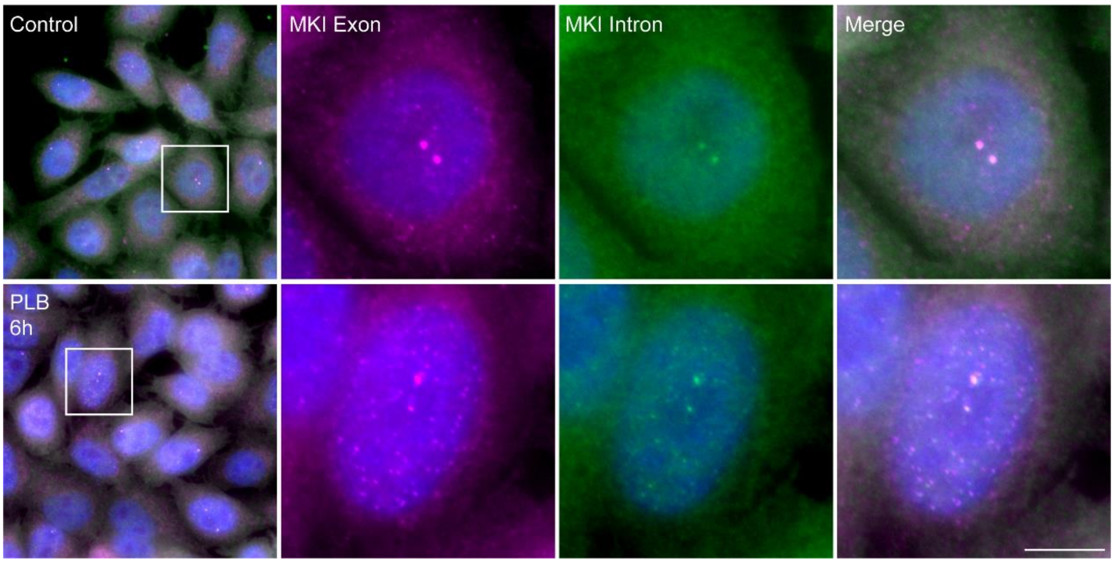

Fig. S4 *MKI* unspliced transcripts appear in the nucleoplasm during splicing inhibition by PLB. No unspliced transcripts were observed in the cytoplasm. *MKI* exon (pink), *MKI* intron (green). Hoechst DNA stain is in blue. Bar = 10 µm.
